# Home Nursing for Insured Persons

**Published:** 1922-09

**Authors:** William G. Blair

**Affiliations:** Secretary, The Reading Council for Nursing Services (and Clerk to the Reading Insurance Committee)


*To the Editor of* THE HOSPITAL AND HEALTH REVIEW.

### Home Nursing for Insured Persons.

*To the Editor of* THE HOSPITAL AND HEALTH REVIEW.

SIR,—With reference to the article on page 277 of the July issue of THE HOSPITAL AND HEALTH REVIEW, on "Home Nursing for Insured Persons," it may interest your readers to know that the Reading Council for Nursing Services has also started a scheme under which payment at a capitation rate is being made by a number of Approved Societies in respect of their members. The scheme provides that, wherever the person may be resident, if he or she can obtain the services of a nurse, the Council will meet the cost. The Council consists of representatives of Approved Societies parties to the scheme, local Nursing Associations and others interested.

Yours faithfully,
WILLIAM G. BLAIR,
Secretary, The Reading Council for Nursing Services (and Clerk to the Reading Insurance Committee).

